# Prediction and molecular field view of drug resistance in HIV-1 protease mutants

**DOI:** 10.1038/s41598-022-07012-x

**Published:** 2022-02-21

**Authors:** Baifan Wang, Yinwu He, Xin Wen, Zhen Xi

**Affiliations:** State Key Laboratory of Elemento-Organic Chemistry and Department of Chemical Biology, Nankai University, Collaborative Innovation Center of Chemical Science and Engineering, Tianjin, 300071 People’s Republic of China

**Keywords:** Biophysical chemistry, Computational chemistry

## Abstract

Conquering the mutational drug resistance is a great challenge in anti-HIV drug development and therapy. Quantitatively predicting the mutational drug resistance in molecular level and elucidating the three dimensional structure-resistance relationships for anti-HIV drug targets will help to improve the understanding of the drug resistance mechanism and aid the design of resistance evading inhibitors. Here the MB-QSAR (Mutation-dependent Biomacromolecular Quantitative Structure Activity Relationship) method was employed to predict the molecular drug resistance of HIV-1 protease mutants towards six drugs, and to depict the structure resistance relationships in HIV-1 protease mutants. MB-QSAR models were constructed based on a published data set of *K*_i_ values for HIV-1 protease mutants against drugs. Reliable MB-QSAR models were achieved and these models display both well internal and external prediction abilities. Interpreting the MB-QSAR models supplied structural information related to the drug resistance as well as the guidance for the design of resistance evading drugs. This work showed that MB-QSAR method can be employed to predict the resistance of HIV-1 protease caused by polymorphic mutations, which offer a fast and accurate method for the prediction of other drug target within the context of 3D structures.

## Introduction

HIV-1 protease (HIV-1 PR) plays important roles in HIV life cycle by cleaving the Gag and Gag-Pol polyproteins to yield individual mature proteins which are essential for maturation of infectious HIV particles^1^. Inactivation of HIV-1 PR causes the production of immature, noninfectious viral particles and hence blocks further HIV infection. HIV-1 PR is a homodimeric aspartic protease and its substrate binding pocket includes the Asp25(25′)-Thr26(26′)-Gly27(27′) catalytic triad and flap regions^2,3^. The active site of HIV-1 protease can be divided into eight subsites S4-S3-S2-S1-S1′-S2′-S3′-S4′ and the eight corresponding substrate residues are denoted as P4-P3-P2-P1-P1′-P2′-P3′-P4′, where the scissile bond is between P1 and P1′ ^2,3^. HIV-1 PR has been a molecular target for structure-based drug design and was proven to be effective^4–6^. Currently there are ten FDA approved protease inhibitors (PIs) developed to date^7^.

HIV is an RNA virus which has a high mutation rate (estimated at 10^−4^ per nucleotide per replication) and a high frequency of recombination^8^. In combined with high replication rate of the virus, HIV can quickly develop resistant strains against PIs^9,10^. As a result, the current inhibitors are becoming less effective against rapidly emerging drug-resistant HIV mutants^11–13^. Hence, the understanding and prediction of resistance against HIV-1 PR mutants is important for the selection of the most adequate antibiotic and antiviral therapy, as well as to develop more effective treatment.

Various computational methods have been developed to understand and predict the drug resistance of HIV-1 PR mutants. Sequence-based methods such as ANRS^14^, HIVdb^15^ and REGA^16^, as well as machine learning-based algorithms such as geno2pheno^17^ and SHIVA^18^ are used to predict the HIV-1 PR drug resistance, which mainly focus on the prediction from genotypes to phenotypes of HIV-1 PR mutants. These sequence-based methods are relatively fast and low cost. An important limitation of these approaches is that mutations are not considered in the context of the three-dimensional structure of the target. Thus, these methods fail to capture the links between the mutations and the mutation-induced structural changes confer to the resistance^19,20^.

Structure-based methods are inherently more suitable to predict and interpret the impact of mutations on target-drug interactions. These methods include using molecular docking to predict resistance of HIV1-PR to different inhibitors^21,22^, using molecular field potential to predict the genotypes of HIV drug resistance^23^, and using molecular dynamics (MD) simulations to study the impact of mutations on structural dynamics, stability and binding affinity^24–28^. Although these methods can provide detailed structural information related to mutational drug resistance, they are not suitable for the large scale prediction of the resistance of mutants against drugs due to being time-consuming and offering limited predictive accuracy.

**Figure 1:**
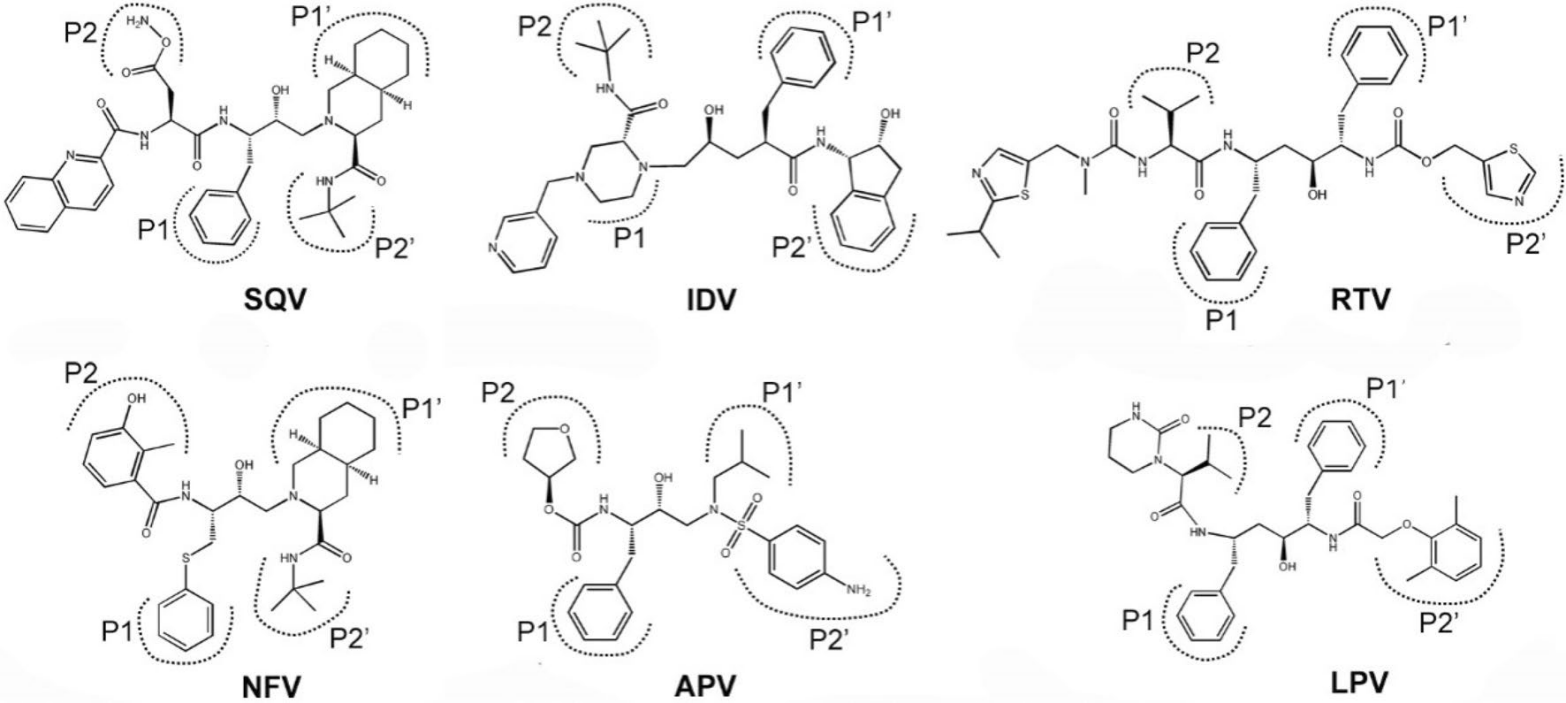
The chemical structures of six FDA approved HIV-1 protease inhibitors. SQV: saquinavir; IDV: indinavir; RTV: ritonavir; NFV: nelfinavir; APV: amprenavir; LPV: lopinavir. P1, P2, P1′, P2′ represent the binding site of substrate residue among the cleavage site of HIV PR.

Previously we have developed a method called as MB-QSAR (Mutation dependent Biomacromolecular Quantitative Structure Activity Relationship), which allows to rapidly predict the drug resistance accurately and supplies sufficient structural information directly related to the drug resistance. MB-QSAR method has been successfully applied on the prediction of the herbicide resistance of Acetohydroxyacid Synthases (AHAS) causing by the single or double mutations^29,30^, as well as the ligand binding affinity to ShHTL7 mutants^31^. Here we extend MB-QSAR method to predict the resistance of HIV-1 PR mutants from real patient sequences containing large numbers of mutations towards six HIV-1 PR inhibitors (SQV, IDV, RTV, NFV, APV and LPV, Fig. 1). We obtained well prediction accuracy for the binding affinity of drugs to HIV PR mutants. The interpretation of these MB-QSAR models revealed the molecular field view of the drug resistance in HIV-1 PR mutants, which provides insight into the understanding of the drug resistance mechanisms and structural information for the design of resistance evading inhibitors. Compared to those methods mentioned above, MB-QSAR method is capable of predicting the binding affinity of PIs to various HIV-1 PR mutants fast and accurately, and providing the structural basis of the drug resistance in HIV-1 PR mutants.

## Results and discussion

**Figure 2:**
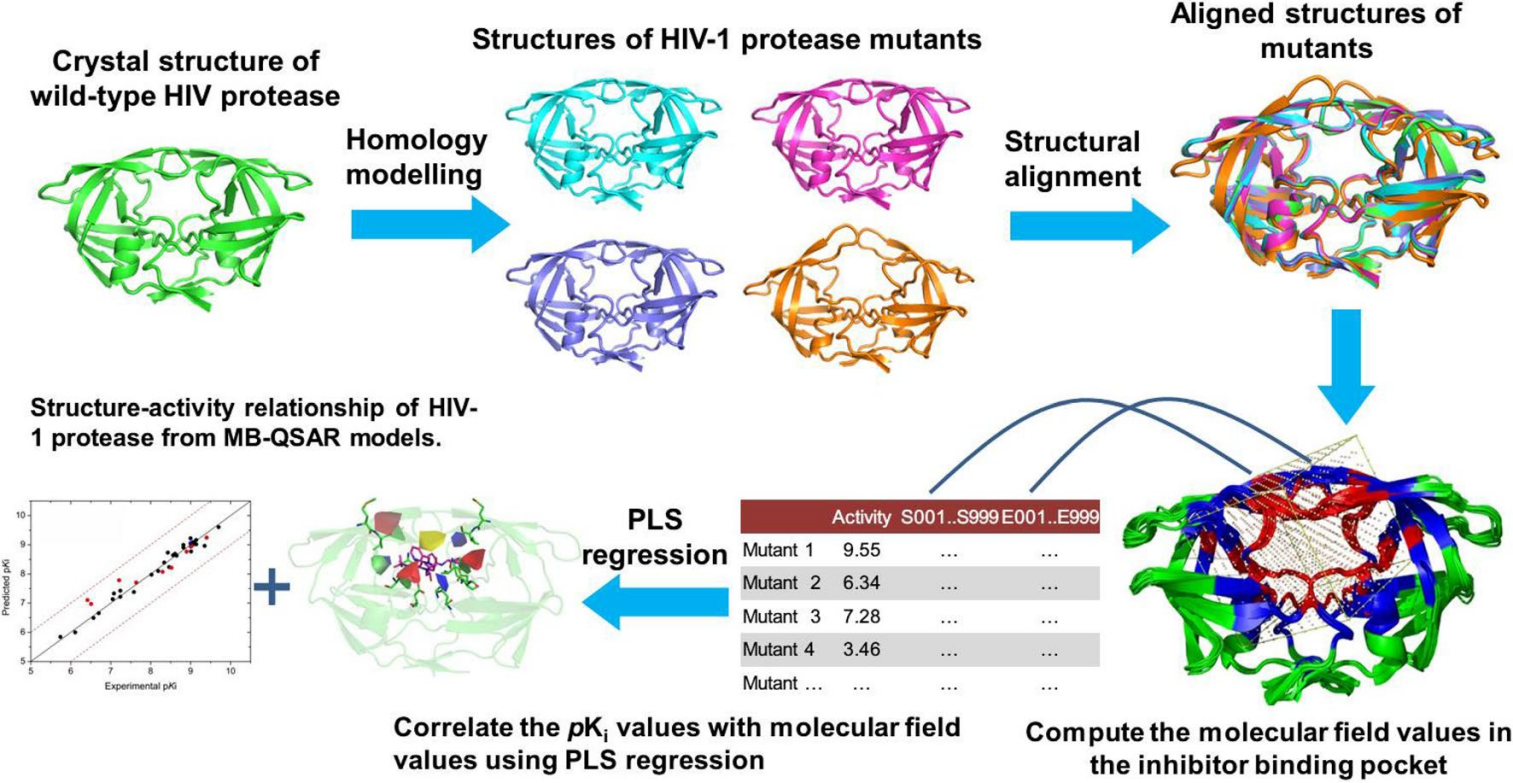
The general flow of MB-QSAR method. The structures of a series HIV-1 PR mutants were constructed and aligned, then the molecular field values in the inhibitor binding pocket were computed using probe atoms. The PLS regression method was used to correlate the p*K*_i_ values and the calculated molecular field descriptors to achieve the MB-QSAR models. Based on the constructed MB-QSAR models, the molecular drug resistance of HIV-1 PR mutants could be predicted, and interpreting the MB-QSAR models could yield molecular field view for the interaction between the inhibitors and the HIV-1 PR mutants.

MB-QSAR method is based on the traditional small molecule 3D-QSAR methodology^32,33^, in which a series of proteins mutants were treated as “analogies” been targeted by the same small molecule. MB-QSAR method assumes that a suitable sampling of the molecular field values in the inhibitor binding pocket of the mutants can yield models which can quantitatively predict the drug resistance of new mutants and provide information to help the understand of the drug resistance mechanisms and the design of resistance evading inhibitors (Fig. 2).

Here we have constructed the MB-QSAR models for six drugs (SQV, IDV, RTV, NFV, APV and LPV) against HIV PR mutants. The statistical results of MB-QSAR/CoMFA models for the six drugs were shown in Table 1. Six statistical parameters, including the *q*^2^, *ONC*, *r*^2^, *SEE*, *F*-value and *r*_*pred*_^2^ value, were obtained to assess the quality of MB-QSAR models. In general, our MB-QSAR/CoMFA models for the six drugs were quite well considering their cross-validated squared correlation coefficient *q*^2^ values were higher than 0.6 using 4 or 3 components and the high *r*^2^ values. The higher *F*-values and the lower *SEE* also indicated our models had higher explanatory power.

**Table 1:** Summary of statistical data for MB-QSAR analyses.

	SQV	IDV	RTV	NFV	APV	LPV
*ONC*^a^	4	4	4	4	4	3
*q*^2^ ^b^	0.609	0.657	0.646	0.623	0.655	0.624
*SEE*^c^	0.205	0.203	0.223	0.171	0.162	0.221
*r*^2^ ^d^	0.962	0.966	0.968	0.961	0.979	0.960
*F*-value^e^	179.264	245.669	265.996	188.851	301.502	234.802
*r*_pred_^2^ ^f^	0.858	0.799	0.825	0.603	0.875	0.736
Contributions^g^						
S	0.567	0.593	0.593	0.577	0.575	0.594
E	0.433	0.407	0.407	0.423	0.425	0.406

^a^*ONC*: optimal number of components. ^b^*q*^2^: cross-validated squared correlation coefficient from leave-one-out (LOO). ^c^*SEE*: standard error of estimate from non-cross-validation. ^d^*r*^2^: square of the correlation coefficient of non-cross-validation. ^e^*F*-value: *F*-test value. ^f^*r*_pred_^2^: square of the correlation coefficient calculated from the test set. ^g^Field contributions: S = steric field, E = electrostatic field.

In these MB-QSAR/CoMFA models, the contributions of the steric and electrostatic fields are approximately 60% and 40%, respectively, which indicated that the steric field plays more important role in the HIV-1 PR mutants confer resistance to PIs. The test sets were used to verify the external predictive power of the models. For all of the MB-QSAR/CoMFA models, the *r*_pred_^2^ values are higher than 0.7 (except for NFV, which has an *r*_pred_^2^ value of 0.603), indicating a high prediction accuracy for all of the MB-QSAR/CoMFA models.

Compared to CoMFA methods, CoMSIA can utilize up to five different molecular fields (steric, electrostatic, hydrophobic, hydrogen bond donor, and hydrogen bond acceptor field) as well as their combinations to construct QSAR models. We tested all 31 possible field combinations to generate the MB-QSAR/CoMSIA models. The field (combinations) display highest *q*^2^ value and external predictive ability (*r*_pred_^2^) were chosen as the MB-QSAR/CoMSIA models for the six drugs (Table S2). These models mostly involved steric and hydrophobic fields (Table S2), indicating an important role of steric interaction in the binding of PIs to HIV PR mutants.

**Figure 3:**
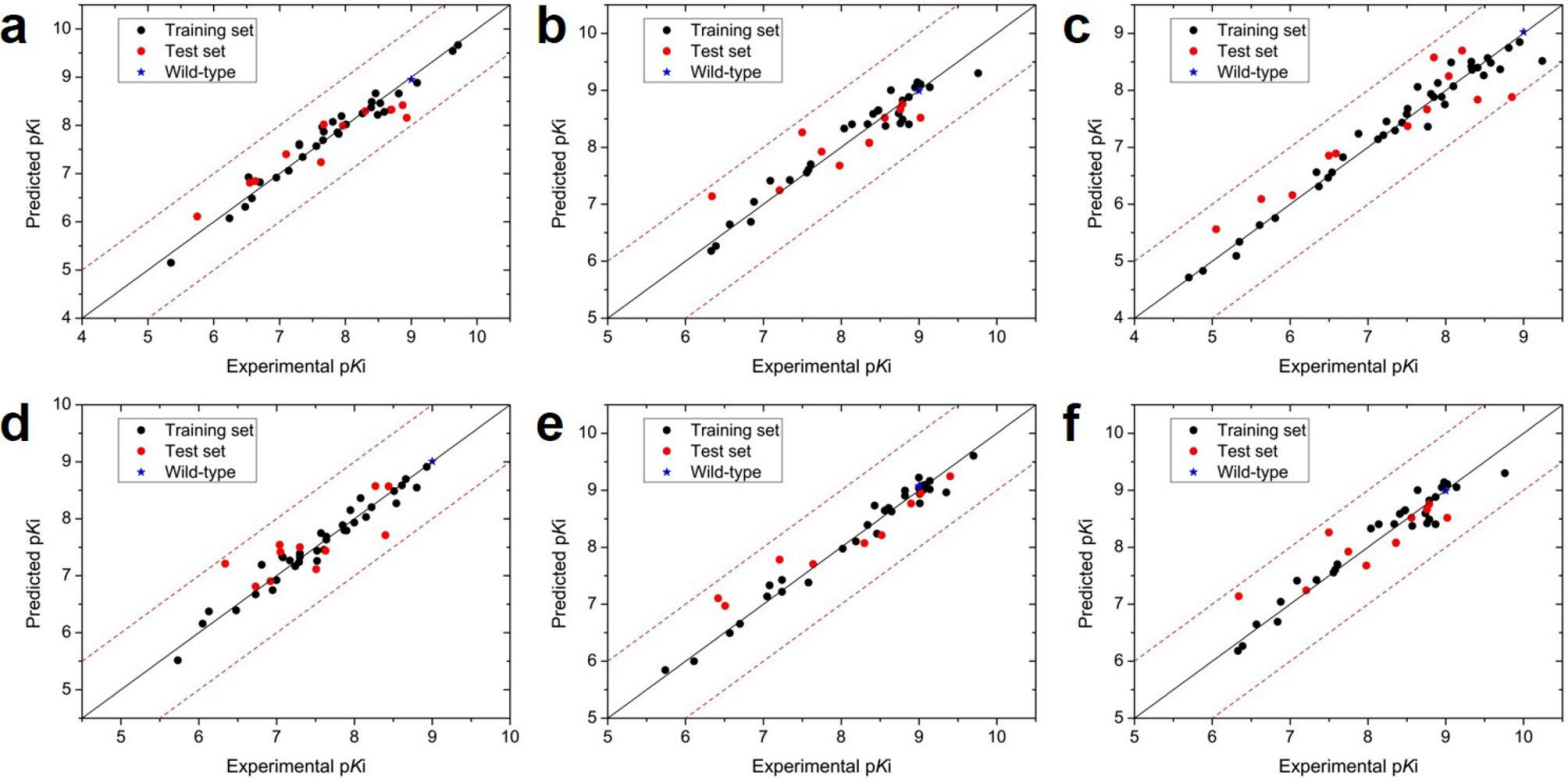
Plots of the experimental and predicted relative p*K*i values in the MB-QSAR COMFA models. (**a**) SQV; (**b**) IDV; (**c**) RTV; (**d**) NFV, (**e**) APV; (**f**) LPV. The values from training and test set are showing in black and red dots, respectively. The black line represents the identity between the experimental and the predicted values, while the red dash lines display one logarithm value error from identity.

The obtained CoMFA and CoMSIA models for the six drugs were used to predict the relative p*K*_i_ value for the test set. As shown in Fig. 3 and Fig. S1, all the errors between the experimental p*K*_i_ values and the predicted p*K*_i_ values in the training and test set are smaller than 1.0 log unit, which indicated the quite well predictive ability of the constructed MB-QSAR models.

**Figure 4:**
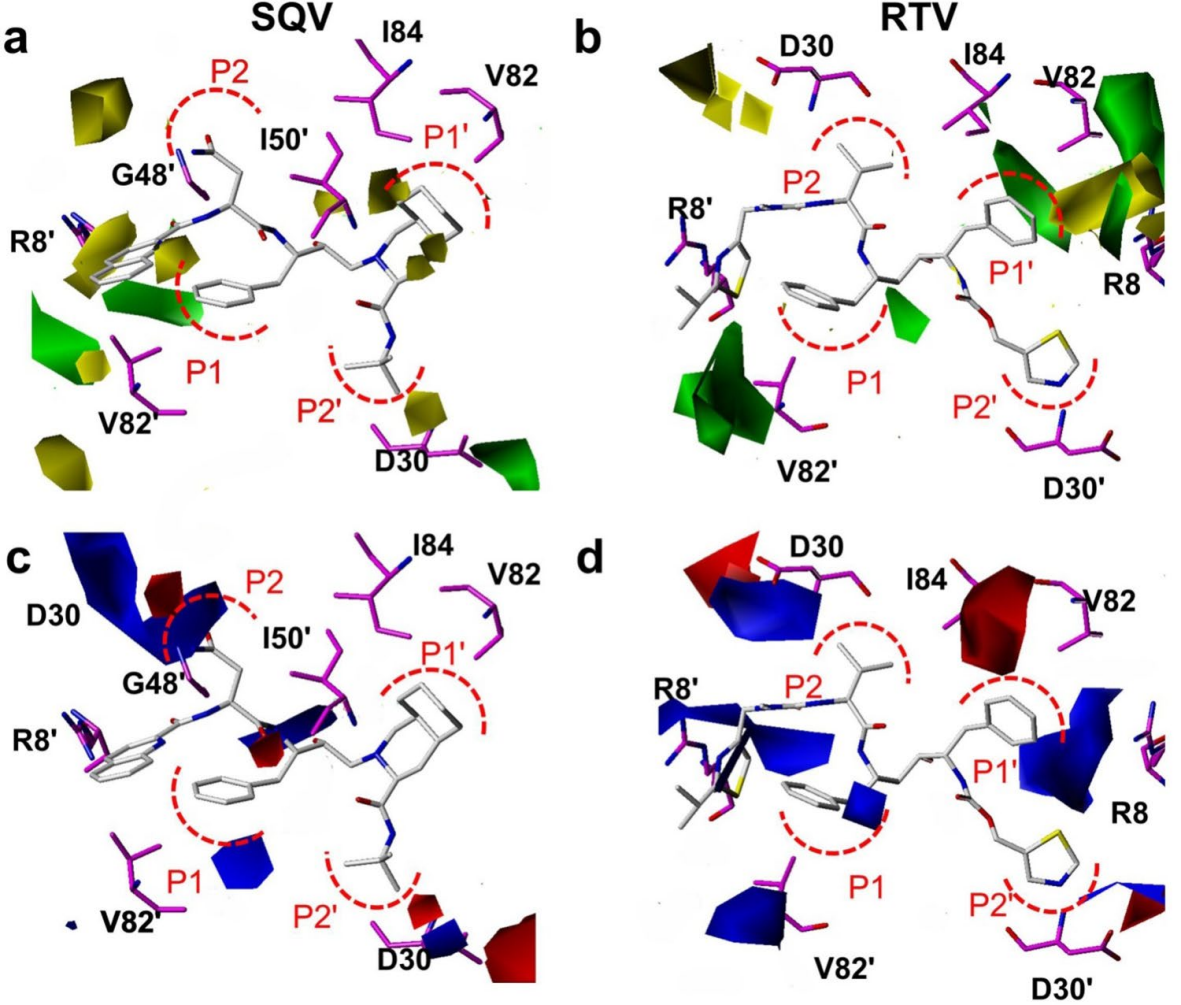
MB-QSAR/CoMFA contour maps of steric (upper panel) and electrostatic (lower panel) fields for SQV and RTV. PIs and representative residues of HIV-1 PR are shown in white and magenta sticks, respectively. Amino acids of HIV PR labeled with apostrophes belonging to another monomer with respect to the ones without apostrophes. Steric effect maps indicated areas where steric interaction was predicted to increase (green) or decrease (yellow) the potency of the p*K*i values for these inhibitors. Electrostatic effect maps indicated where high charge density (negative charge, red) and low charge density (positive charge, blue) regions were expected to increase the potency of the p*K*i values for these inhibitors.

Here we derived the 3D coefficient contour maps of CoMFA models which can show the structural impacts on the binding of drugs, thus can provide a view of the drug resistance mechanisms (Fig. 4 and Fig. S2). The contours were mapped on the structure of wild-type HIV PR complexed with inhibitors. The CoMFA steric interactions are represented by green and yellow contours, while CoMFA electrostatic interactions are shown with red and blue contours. The bulky substituents in HIV PR are favorable in the green regions of steric contours for enhancing the inhibitory activity, while those in yellow regions may lead to a decrease in inhibitory activity. Meanwhile, in the map of the electrostatic field, the blue contours indicate that electropositive charges in HIV PR are favored for inhibitory activity, while the red contour designates an increase in inhibitory activity of the electronegative charges.

As shown in Fig. 4 and Fig. S2, the yellow contours surrounding the residueGly48 and Gly48′ in the complex of HIV PR with SQV, IDV, NFV and LPV indicated that the steric interactions were not favorable for the binding of PIs, which are consistent with the fact that introduction bulky residue in this site can caused resistance to PIs^34^. The Gly48 of HIV-1 PR mainly interacts with these inhibitors through the interaction between its backbone atoms (Fig. S3). The introduction of bulky residues such as valine and methionine in this site can disrupt the interaction between the backbone of Gly48 and PIs thus introduce the resistance to these drugs. The green and yellow contours between residue of Val82 and PIs, indicating that the mutations of Val82 to different residues can introduce favorable and unfavorable steric interaction towards different PIs, such as V82F mutation should be unfavorable for the binding of SQV and RTV, while the V82F or V82L is unfavorable for the binding of NFV and APV, respectively, which again agreed with these mutations can cause resistance to the corresponding drugs. Residue of Ile50 and Ile84 also involved in the HIV-1 PR resistance mutations. The yellow contours between Ile50 and SQV, NFV and IDV; as well as between Ile84 and SQV, IDV and NFV, indicated that the mutations of Ile50 and Ile84 to larger residues caused steric effect can confer the resistance of HIV-1 PR towards the above mentioned PIs.

In the electrostatic fields (Fig. 4 and Fig. S2), red and blue contours were found between residues Asp30 and several PIs (SQV, RTV, APV, LPV and IDV), indicating that negative and positively charge may contribute to the binding of PIs, which indicated that mutation of Asp30 such as D30N mutation can increase or decrease the binding PIs (Table S3). It is interesting to find that, there are blue contours between Arg8 and two PIs (RTV and APV). This may suggest that the positive charge of the Arg8 is essential for the binding of RTV and APV to HIV-1 PR. Although Arg8 is not involved in the drug resistant mutation of HIV-1 PR, the orientation of the side chain of Arg8 is affected by the mutation of other residues, which resulted in the change of molecular field around this residue.

The contour maps derived from the MB-QSAR models also provide information for the design of resistance evading inhibitors towards HIV-1 PR mutants. The yellow contours between the HIV-1 PR and the P1 site of several PIs (SQV, IDV, NFV, APV and LPV, Fig. 4and Fig. S2) indicate that the substitutions of phenyl group with small groups might increase the resistance evading abilities for the PIs. In the meantime, green contours appear between HIV-1 PR and P1' site of PIs (SQV, NFV and RTV) suggest that increasing the steric interaction for this site and HIV-1 PR can result in better resistance evading abilities. While for APV, a smaller group is favorable to interact with the P1′ site of HIV PR mutants. In the electrostatic filed, the blue contours between Asp30 and the P2 or P2' site of PIs, indicated that a positive charged substitution at P2 site should increase the binding of PIs towards HIV-1 PR mutants. In summary, analyzing the contour maps in HIV-1 PIs yielded many clues for the design of resistance evading inhibitors for HIV-1 PR mutants.

The PI-resistant mutations of HIV-1 PR have been classified into major and minor mutations depending on their effect in antiviral therapy^34^. Our MB-QSAR study of HIV-1 PR involved 59 HIV-1 PR variants, which cover most of the major and minor mutation sites (Fig. S4). The major mutation sites are mostly located in the active site to directly interact with substrate or inhibitors, while the minor mutation sites are the residues mostly located outside of the active site. The minor mutations can influence the binding of the inhibitors or substrates through perturbations of the active site by the transmitted conformational effects. The real patient sequence of HIV-1 PR usually contain multiple major and minor mutations, thus the resistance of HIV-1 PR mutant in patients to PIs are conferred by both major and minor mutations. It can be seen from Fig. S5 that variants with major and minor mutation would cause structural change in the HIV PR, especially on the “flap” region that plays essential role on the binding of inhibitors. In the meantime, these mutations also caused the change on the electrostatic potential of the HIV PR (Fig. S5b and c). The effect caused by the mutations on the binding of PIs to HIV-1 PR mutants are regards as the change of the molecular field values in the active site in HIV-1 PR mutants in our MB-QSAR studies. We showed that our MB-QSAR method could be employed to accurately predict the resistance of HIV mutants to PIs caused by both major and minor mutations.

Currently there are two categories of method were developed to predict the resistance of HIV-1 PR mutants to inhibitors: sequence based and structure based methods. The sequence based method represents a fast prediction method, however the resistance caused by the mutation is not considered in the context of the 3D structure of the target protein. The structure-based methods, such as molecular dynamics simulation, are computationally expensive. To build the MB-QSAR model fot the HIV-1 PR mutants to inhibitors, it only took several hours to run the program on a desktop computer with a Intel(R) Core(TM)i5-8250 CPU. Our work thus presents a fast, structure based method capable of accurately predict the binding affinity of PIs to various HIV-1 PR mutants.

## Conclusions

It is a great challenge to find the “perfect” drugs to conquer the drug resistance against HIV. In this work, the MB-QSAR method was employed to predict and provide a molecular field view of drug resistance in HIV-1 protease mutants. Reliable MB-QSAR models were constructed for six drugs (SQV, IDV, RTV, NFV, APV and LPV) with accurate prediction abilities for the prediction of drug resistance for a series of HIV-1 PR mutants. The relationships between the structures of protease mutants and the drug resistance were derived from these models. Interpretation of the relationships supplies important structure information will benefit the understanding of the HIV-1 PR mutational drug resistance mechanism, and provide important clues for the design of efficient resistance-evading inhibitors as well as help to rationalize and personalize the therapeutic decision-making process. Considering that the problem of mutation-induced resistance cuts across virtually all infectious diseases, we believe our method may be extended to a wide range of drug targets besides HIV.

## Materials and methods

### Biological data

The inhibition constant values (*K*_i_) of six HIV PIs to different HIV-1 PR mutants were taken from research groups of Dunn^35–38^ and Konvalinka^39–44^. A total of 60 HIV-1 PR variants including the wild type and mutants were listed in Table S1. The *K*_i_ values of mutants were converted to relative *K*_i_ against the wild-type and subsequently treated to relative p*K*_i_ values and calibrated with an artificial number of 8.0 to make the relative p*K*_i_ value of wild-type to 9.0 (relative p*K*_i_ = log(relative *K*_i_) + 8.0).

### Modeling of HIV-1 PR mutant structures

The crystal structures of wild-type and mutant HIV-1 PR were taken from RCSB database if available. For the other HIV-1 PR mutants the structures were constructed via homology modeling using the SWISS-MODEL Protein Modeling Server^45^. The inhibitors were then placed in the mutant structures according to their coordinates in the wild-type PR. A total of 279 complex structures were constructed (SQV: 44, IDV: 53, RTV: 52, NFV: 47, APV: 40, LPV: 43). The hydrogen atoms were added to these structures by SYBYL6.9 (The Asp25 and Asp25′ were protonated according to their bound inhibitor: Asp25 was protonated when bound to SQV, IDV or LPV; Asp25′ was protonated when bound to RTV, NFV or APV, respectively^46^.) The Gastger-Mashii charges were assigned to small molecules and the amber charges were assigned to proteins. The complex structures were first minimized for 1000 times using the Tripos force field and the Powell method. Then the inhibitor and residues within 8 Å from the inhibitor were minimized to a 0.01 kcal/(mol*Å) convergences. The residues which were 8–16 Å from the inhibitor were kept rigid and considered the interactions with the interesting region residues and the other residues 16 Å away from the inhibitor were ignored during the second minimization process.

### MB-QSAR modeling

Prior to MB-QSAR modeling, the structures were aligned with the backbone atoms of the residues in the inhibitor binding pocket within 3–6 Å away from the inhibitor with respect to the one of wild-type, according to our previous studies^29,30^. The atom by atom least-square fit was used in the alignment. The inhibitors were removed from the complex structures after alignment.

For each of drugs, the proteins were divided into training set and test set. MB-QSAR models were constructed based on the training set. The test set was used to evaluate the external predictively of these models. Special cares were taken to ensure the appropriate ranges and distributions of the p*K*_i_ values for training and test set, respectively.

To calculate the molecular field values, the lattice were centered on the inhibitors with the edge extended 4 Å away from the edge of inhibitor and a grid spacing of 2 Å. The CoMFA fields were calculated with a distance-dependent dielectric constant (1/*r*), and a sp^3^ carbon atom with + 1.0 charges serving as the probe atom were used to calculate the steric and the electrostatic field values. An energy cutoff value of 30 kcal/mol was used for both the steric and electrostatic fields. In CoMSIA studies, five indices (steric (S), electrostatic (E), hydrophobic (H), hydrogen-bond donor (D) and hydrogen-bond acceptor (A) descriptors) were calculated with the same lattice as in the CoMFA fields calculation, using the probe atom with a radius of 1.0 Å, a charge of + 1.0 and a unit hydrophobicity value. A Gaussian-type distance dependence function was used between the grid points and atoms of the proteins.

The CoMFA and CoMSIA field values were used as independent variables, while the relative p*K*_i_ values were used as dependent variables in the partial least squares (PLS) regression analyses to derive the MB-QSAR models. The cross-validation with the leave-one-out (LOO) option was carried out and the SAMPLS method was used in CoMSIA to obtain the optimal number of components (*ONC*), and the *ONC* was used to generate the PLS regression models by non-cross-validated analysis. In the case of CoMSIA analysis, 31 analyses were carried out using the five fields separately and in all possible combinations. All the QSAR calculations were done in SYBYL6.9.

## Supplementary Information

Supplementary Information.
