# Supplementary material for: Prediction and molecular field view of drug resistance in HIV-1 protease mutants
Source: Sci Rep. 2022 Feb 21;12:2913. doi: 10.1038/s41598-022-07012-x (PMC8861105; doi:10.1038/s41598-022-07012-x)
Supplement: Supplementary file 1 — Supplementary Information. [file 41598_2022_7012_MOESM1_ESM.pdf]

Supporting Information

Prediction and Molecular Field View of Drug Resistance in HIV-1 Protease Mutants

Baifan Wang, Yinwu He, Xin Wen*, and Zhen Xi*

State Key Laboratory of Elemento-Organic Chemistry and Department of Chemical Biology, Nankai University. Collaborative Innovation Center of Chemical Science and Engineering, Tianjin 300071, P. R. China. Correspondence and requests for materials should be addressed to X.W. (email: xinwen@nankai.edu.cn) or Z.X. (email: zhenxi@nankai.edu.cn)

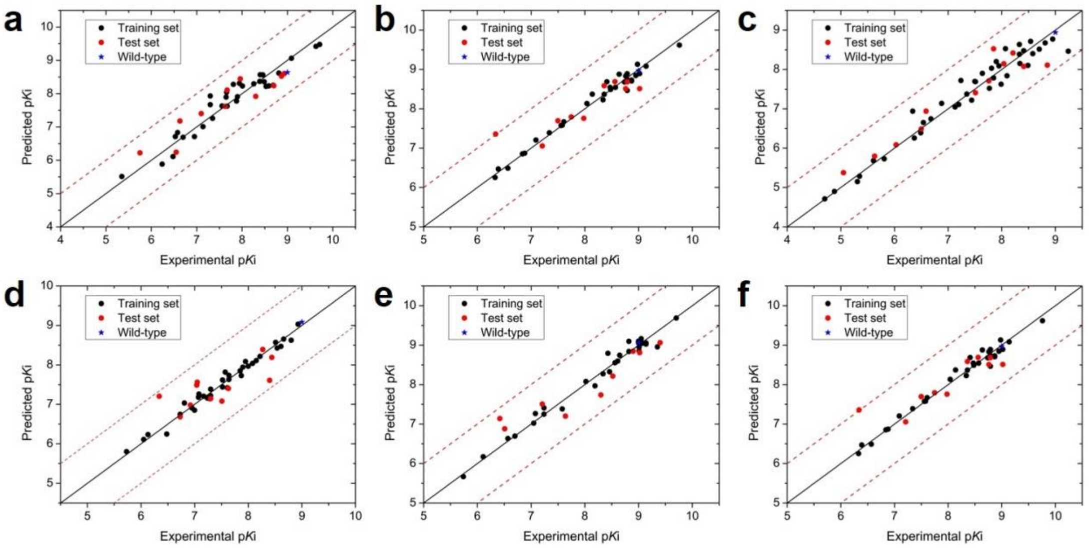

Figure S1. Plots of the experimental and predicted relative p*K*i values in the MB-QSAR COMSIA models. (**a**): SQV; (**b**): IDV; (**c**): RTV; (**d**): NFV, (**e**): APV; (**f**): LPV. The values from training and test set are showing in black and red dots, respectively. The black line represents the identity between the experimental and the predicted values, while the red dash lines display one logarithm value error from identity.

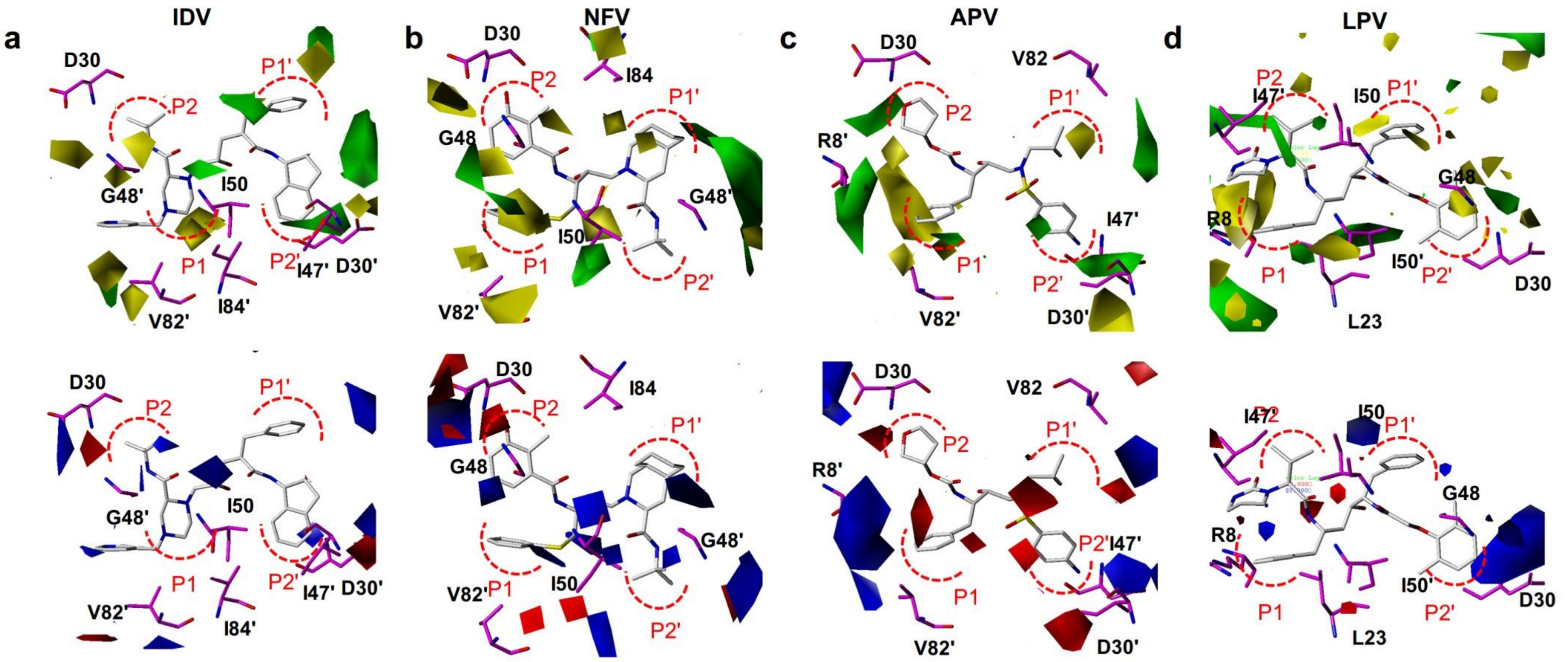

Figure S2. MB-QSAR/CoMFA contour maps of steric (upper panel) and electrostatic (lower panel) fields for IDV (a), NFV (b), APV (c) and LPV (d). PIs and representative residues of HIV-1 PR are shown in white and magenta sticks, respectively. Steric effect maps indicated areas where steric interaction was predicted to increase (green 80%) or decrease (yellow 20%) the potency of the p*K*i values for these inhibitors. Electrostatic effect maps indicated where high charge density (negative charge) (red 20%) and low charge density (positive charge) (blue 80%) regions were expected to increase the potency of the p*K*i values for these inhibitors.

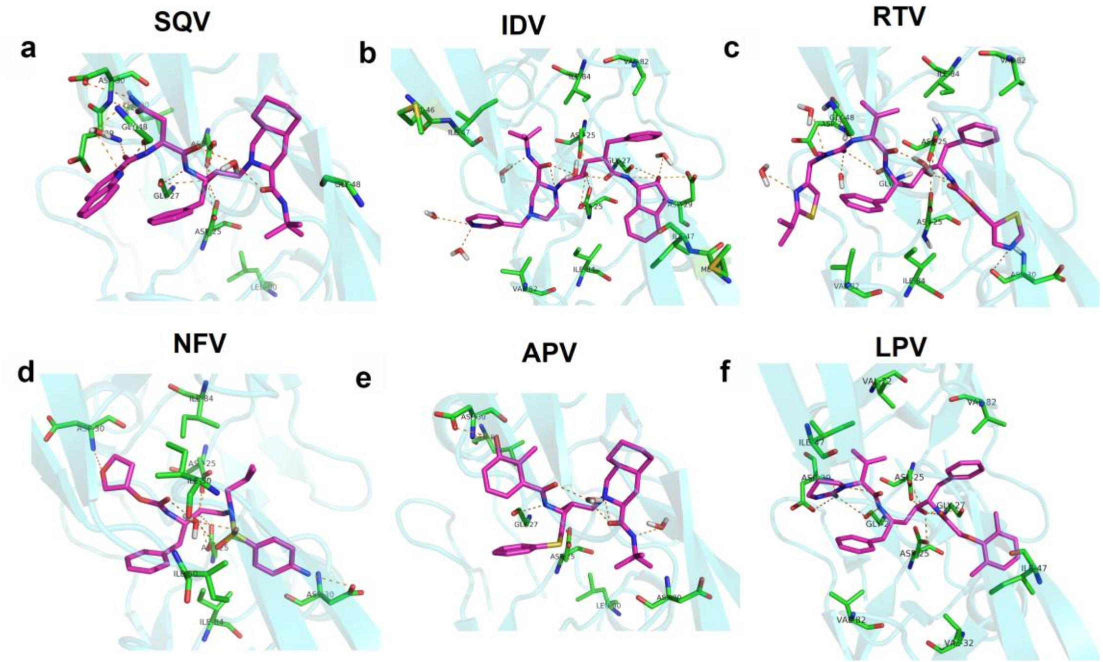

Figure S3. The binding of protease inhibitors to HIV PRs. The PIs are showing in magenta sticks, while the residues of HIV PR are showing as green sticks.

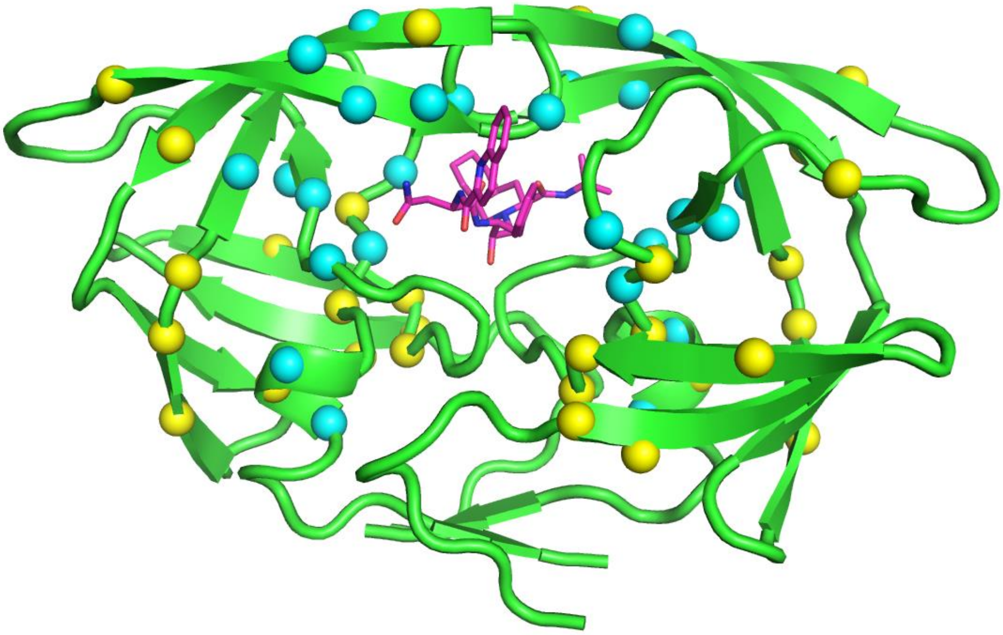

Figure S4. The major and minor PI-resistant mutation of HIV1-PR. The major mutation sites are showing in cyan spheres; while the minor mutation sites are showing in yellow spheres, the SQV is showing in magenta sticks. The major mutations are: D30N, V32I, L33F, M46IL, I47VA, G48VM, I50VL, I54VTALM, L76V, V82AFTSL, I84V, N88SD, and L90M. The minor mutations are: L10FIVRY, V11IL, K20RIMTV, L23I, L24IFM, M36I, K43T, M46V, G48ASTQL, F53LY, I54ST,

Q58E, A71VTIL, G73STCADV, T74PS, V82MC, N83DS, I84AC, I85V, N88TG, and L89VT.

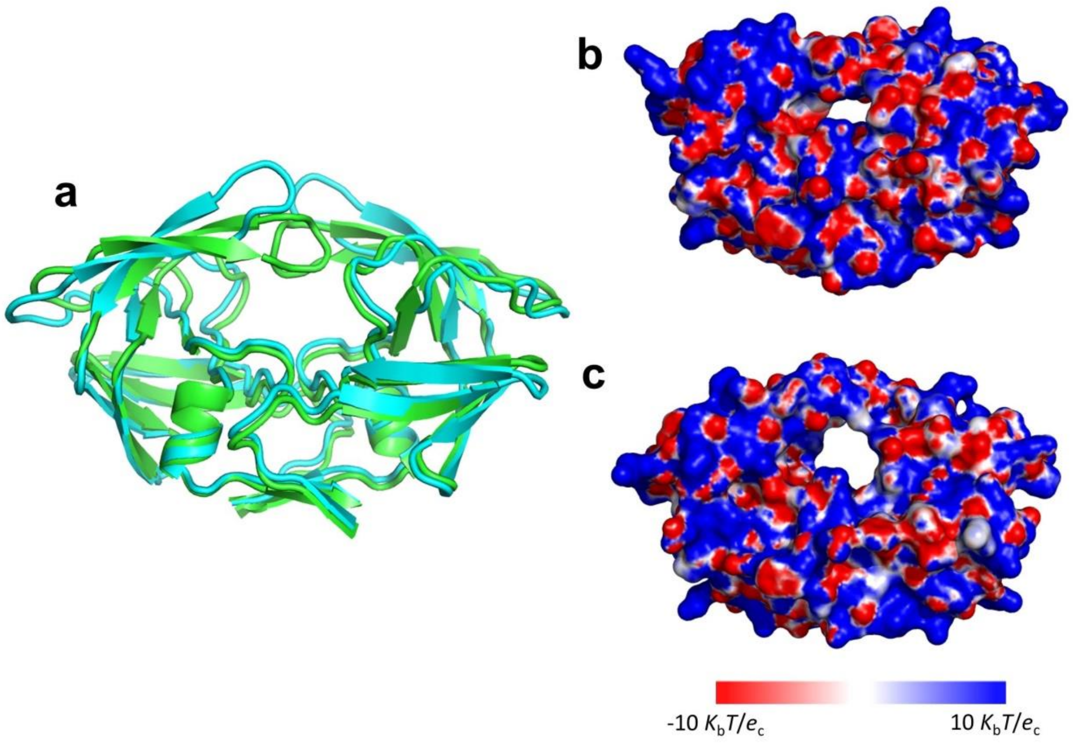

Figure S5. Comparison of the structure of wild-type HIV PR and its mutants. (a): The overlap of structure of wild-type HIV PR (green) and its mutant (M27, see Table S1, cyan). Both structures are showing in cartoon model. The "flap" region was highlighted. (b) The surface model of wild-type HIV PR mapped with electrostatic potential. (c) The surface model of HIV PR mutant mapped with electrostatic potential.

Table S1. HIV PR variants use in this work.

Label	HIV PR variants
M01	A71V
M02	A71V, V82T, I84V
M03	D30N
M04	D30N, A71V
M05	D30N, L63P, A71V, L90M
M06	D30N, L90M
M07	D30N, N88D
M08	D30N, N88D, L90M
M09	E35D, N37S, L63P, I72V
M10	G48V, L90M
M11	I3V, I13V,E35D,M36I,S37N,R41K,H69K,L89M
M12	I3V, I13V,E35D,M36I,S37N,R41K,H69K,V82F,L89M
M13	I13V, K20R, V32I, L33F, E35D, M36I,R41K, K43T, I47V, I54M, I62V, L63V,A71V, I72T, G73S, T74P, V82L, L89V,I93L
M14	I47A
M15	I47A, I54V
M16	K20R, V32I, L33F, M36I, I54V, L63P, A71V, V82A, I84V, L90M
M17	K20R, V32I, L33F, M36I, I54V, L63P, A71V, V82A, L90M
M18	K20R, V32I, L33F, M36I, L63P, A71V, V82A, I84V, L90M
M19	K20R, V32I, L33F, M36I, L63P, A71V, V82A, L90M
M20	K20R, V32I, L33F, M36I, M46I, I54V, L63P, A71V, V82A, I84V, L90M
M21	K20R, V32I, L33F, M36I, M46I, L63P, A71V, V82A, I84V, L90M
M22	K20R, V32I, L33F, M36I, M46I, L63P, A71V, V82A, L90M
M23	K45I
M24	L10F, I13V, D30N, K45I, I50L, L63P, A71I,V77I, N88D
M25	L10F, I13V, G16A, K20M, V32I, E35EE, K43T, M46V, I47V, I54M, I64V, A71V, V82A
M26	L10F, I13V, G16A, K20M, V32I, K43T, M46V, I47V, I54M, I64V, A71V, V82A
M27	L10F, L19I, K20R, L33F, E35D, M36I, R41K, F53L, I54V, L63P, H69K, A71V, T74P, I84V, L89M, L90M, I93L
M28	L10I, I13V, G16E, L33F, M36L, N37T, P39S, K45R, M46L, I54V, K55R, I62V, L63P,A71V, G73D, V82T, I84V, L89V, L90M,I93L
M29	L10I, I13V, I15V, K20R, L33F, E34N, E35D,M36I, K43T, I47V, I50L, F53L, Q58E,I62V, L63P, I66F, A71V, V77I, V82A,I84V, L89V
M30	L10I, I13V, K14R, V32I, L33F, K43T, M46I, I47V, I54L, I62V, L63P, A71T, I72T,G73T, V77I, P79S, I84V, L90M
M31	L10I, I13V, K20I, L33LL, M36I, M46I,I54V, K55R, Q58E, I62V, L63P, C67F, A71V, G73S, V82A, L90M

M32	L10I, I13V, K20I, M36I, M46I, I54V, K55R, Q58E, I62V, L63P, C67F, A71V, G73S, V82A, L90M
M33	I3V, L10I,I13V,L33I,E35D,M36I,S37N,R41K,I54V,L63H,H69K,L89M
M34	I3V, L10I,I13V,L33I,E35D,M36I,S37N,R41K,I54V,L63H,H69K,V82F,L89M
M35	L10I, I15V, E35D, N37S, R41K, I62V, L63P, A71V, G73S, L90M
M36	L10I, L24I, L33F, M46L, I47A, I54V, L63P, A71V, V82A, I84V
M37	L10I, L24I, L33F, M46L, I54V, L63P, A71V, V82A, I84V
M38	L10R, M46I, A71V, V82T, I84V
M39	L63P, A71V, V82T, I84V
M40	L90M
M41	M46I, A71V, V82T, I84V
M42	M46L, I54V, K55R, D60E, Q61E, I62V, L63P, A71V, I72V, V82A, L90M, I93L
M43	M46L, L63P, A71V, L90M, I93L
M44	N88D
M45	N88D, L90M
M46	T12E, K20R, M36I, I62V, L63P, A71V, V82A, L90M, I93L
M47	T12S,I15V,L19I,D30N,M36I,S37A,H69K,L89M,I93L
M48	T12S,I15V,L19I,D30N,M36I,S37A,H69K,L89M,L90M,I93L
M49	T12S,I15V,L19I,D30N,M36I,S37A,H69K,N88D,L89M,I93L
M50	T12S,I15V,L19I,D30N,M36I,S37A,H69K,N88D,L89M,L90M,I93L
M51	T12S,I15V,L19I,M36I,S37A,H69K,L89M,I93L
M52	T12S,I15V,L19I,M36I,S37A,H69K,L89M,L90M,I93L
M53	T12S,I15V,L19I,M36I,S37A,H69K,N88D,L89M,I93L
M54	T12S,I15V,L19I,M36I,S37A,H69K,N88D,L89M,L90M,I93L
M55	T12V, I13V, I15V, K20M, V32I, L33F, K43T,I54L, K55N, I62V, L63P, A71V, I72V,G73S, V77I, V82L, I84V, L89V, L90M
M56	V32I, I47A
M57	V82A
M58	V82F
M59	WT

Table S2. The results of the MB-QSAR/CoMSIA models for the six drugs

	SQV	IDV	RTV	NFV	APV	LPV
ONC[a]	4	5	4	6	4	5
q^{2b}	0.601	0.609	0.689	0.604	0.572	0.602
SEE[c]	0.291	0.178	0.274	0.130	0.206	0.137
r^{2d}	0.925	0.974	0.952	0.979	0.966	0.986
F-value[e]	85.902	258.814	173.595	223.528	184.632	379.544
r^2_{pred}[f]	0.842	0.818	0.910	0.561	0.829	0.758
Contributions[g]						
S	0.328		0.481	0.463	1.000	0.449
H	0.672	1.000	0.519	0.537		0.551

[a]*ONC*: optimal number of components. [b]q^2: cross-validated squared correlation coefficient from leave-one-out (LOO). The q^2 values in CoMSIA models were achieved using SAMPLS method. [c]*SEE*: standard error of estimate from non-cross-validation. [d]r^2: square of the correlation coefficient of non-cross-validation. [e]*F*-value: *F*-test value. [f]r^2_{pred}: square of the correlation coefficient calculated only from the test set. [g]Field contributions: S = steric field, H = hydrophobic field.

Table S3. The experimental and predicted pK_i values by MB-QSAR models.

	Exp. pK_i	Pred. pKi (CoMFA)	Pred. pKi (CoMSIA)		Exp. pK_i	Pred. pKi (CoMFA)	Pred. pKi (CoMSIA)
Training set				Training set			
SQV_WT	9.00	8.95	8.64	IDV_WT	9.00	8.90	9.12
SQV01	8.46	8.66	8.56	IDV02	6.98	7.18	6.96
SQV02	6.53	6.92	6.71	IDV03	8.45	8.57	8.38
SQV04	8.81	8.66	8.62	IDV04	8.11	8.14	8.11
SQV05	7.94	8.19	8.31	IDV06	8.11	8.15	8.09
SQV06	7.90	7.82	7.91	IDV07	8.19	8.30	8.30
SQV07	8.01	8.02	8.23	IDV09	8.29	7.97	8.33
SQV09	8.40	8.49	8.57	IDV11	8.42	8.19	8.33
SQV11	9.09	8.88	9.06	IDV12	7.22	7.33	7.36
SQV12	9.71	9.66	9.48	IDV14	6.90	6.81	6.87
SQV15	7.88	7.85	7.77	IDV15	6.26	6.35	6.35
SQV25	6.58	6.49	6.83	IDV16	5.63	6.07	5.94
SQV31	6.70	6.82	6.69	IDV18	6.79	6.43	6.32
SQV33	7.66	7.69	8.04	IDV19	7.42	7.29	7.32
SQV36	6.24	6.07	5.88	IDV21	7.46	7.54	7.48
SQV37	5.35	5.15	5.51	IDV22	5.96	6.18	5.99
SQV39	6.95	6.92	6.71	IDV23	8.78	8.76	8.69
SQV40	7.81	8.07	8.28	IDV25	6.14	6.15	6.13
SQV41	6.48	6.31	6.11	IDV27	6.56	6.76	6.49
SQV42	7.14	7.06	7.01	IDV31	5.34	5.21	5.19
SQV43	7.56	7.57	7.63	IDV34	6.05	6.25	6.17
SQV44	8.69	8.32	8.25	IDV35	7.34	7.29	7.36
SQV45	7.65	7.97	7.90	IDV36	5.22	5.11	5.37
SQV46	7.35	7.34	7.26	IDV37	6.41	6.08	6.18
SQV47	8.39	8.37	8.37	IDV40	8.46	8.33	8.28
SQV49	8.53	8.46	8.22	IDV41	6.75	6.86	6.93
SQV50	7.67	7.87	7.62	IDV42	7.34	7.24	7.41
SQV52	8.49	8.22	8.35	IDV43	8.22	8.42	8.25
SQV53	8.59	8.28	8.23	IDV44	8.38	8.22	8.09
SQV54	7.30	7.59	7.93	IDV45	8.01	7.81	7.83
SQV56	8.26	8.25	8.29	IDV47	8.24	8.50	8.62
SQV57	7.30	7.61	7.67	IDV48	8.49	8.30	8.30
SQV58	9.63	9.54	9.42	IDV49	8.57	8.54	8.50
Test set				IDV50	8.35	8.37	8.23
SQV03	8.87	8.42	8.53	IDV51	8.74	8.57	8.68
SQV08	7.67	8.02	8.11	IDV53	7.88	7.83	7.94
SQV14	8.70	8.33	8.24	IDV54	7.56	7.71	7.69
SQV26	7.63	7.24	7.62	IDV56	6.33	6.19	6.40
SQV27	5.75	6.11	6.22	IDV57	7.56	8.05	7.93
SQV32	6.63	6.84	7.18	IDV58	8.18	8.15	8.21

SQV34	7.96	7.99	8.44	Test set			
SQV35	7.10	7.40	7.40	IDV01	8.33	8.48	8.28
SQV38	6.55	6.81	6.24	IDV05	8.15	7.74	7.82
SQV48	8.30	8.29	7.92	IDV08	8.56	8.25	8.25
SQV51	8.93	8.15	8.60	IDV10	7.23	7.22	7.62
				IDV17	6.66	6.43	6.16
				IDV20	5.79	6.08	5.95
				IDV26	6.60	6.52	6.71
				IDV32	6.07	5.44	5.37
				IDV33	7.63	7.13	7.09
				IDV38	6.72	7.61	6.41
				IDV39	6.96	7.30	6.81
				IDV46	7.62	7.40	7.81
				IDV52	8.59	8.38	8.48
Training set				Training set			
RTV_WT	9.00	9.02	8.94	NFV_WT	9.00	9.01	9.08
RTV01	8.49	8.26	8.10	NFV01	8.54	8.27	8.43
RTV02	7.20	7.21	7.11	NFV03	7.64	7.63	7.73
RTV03	8.81	8.74	8.67	NFV04	7.00	6.92	6.85
RTV04	8.95	8.85	8.77	NFV07	6.81	7.19	7.03
RTV07	8.33	8.50	8.63	NFV08	7.29	7.24	7.14
RTV11	8.54	8.57	8.71	NFV09	7.57	7.75	7.82
RTV12	7.35	7.29	7.38	NFV10	7.64	7.68	7.63
RTV14	8.70	8.37	8.51	NFV11	8.93	8.91	9.03
RTV15	7.81	7.94	8.03	NFV12	8.66	8.70	8.65
RTV16	4.88	4.83	4.90	NFV16	6.13	6.37	6.24
RTV17	5.81	5.76	5.73	NFV17	7.87	7.80	7.73
RTV19	6.37	6.31	6.26	NFV18	7.85	7.89	7.85
RTV21	5.31	5.09	5.15	NFV20	6.48	6.39	6.25
RTV23	8.33	8.42	8.38	NFV22	7.17	7.27	7.20
RTV25	6.54	6.56	6.65	NFV26	6.73	6.67	6.75
RTV26	7.51	7.68	7.69	NFV31	6.05	6.16	6.11
RTV31	5.35	5.34	5.29	NFV32	6.95	6.75	6.91
RTV32	6.49	6.46	6.39	NFV33	8.00	7.93	7.96
RTV33	7.77	7.36	7.52	NFV34	7.07	7.34	7.18
RTV34	6.34	6.56	6.94	NFV35	7.52	7.44	7.44
RTV35	6.68	6.83	6.74	NFV37	5.73	5.52	5.80
RTV36	4.70	4.72	4.71	NFV38	7.24	7.17	7.16
RTV37	5.61	5.63	5.68	NFV39	7.30	7.39	7.38
RTV39	6.88	7.24	7.14	NFV40	8.22	8.20	8.21
RTV40	8.34	8.36	8.15	NFV41	7.30	7.33	7.22
RTV42	7.44	7.43	7.22	NFV42	7.52	7.26	7.62
RTV43	7.64	8.06	7.90	NFV44	8.61	8.58	8.47
RTV44	8.58	8.48	8.40	NFV45	8.15	8.03	8.11

RTV45	7.99	7.75	7.62	NFV46	7.90	7.79	7.94
RTV46	7.13	7.14	7.05	NFV49	7.61	7.46	7.42
RTV47	8.07	8.49	8.53	NFV50	7.08	7.32	7.26
RTV48	9.24	8.52	8.46	NFV51	8.80	8.55	8.62
RTV49	8.41	8.40	8.48	NFV52	8.51	8.48	8.57
RTV50	7.50	7.58	7.70	NFV54	8.08	8.36	8.04
RTV52	7.95	7.88	8.09	NFV57	7.95	8.15	8.09
RTV54	7.85	7.88	7.78	Test set			
RTV56	7.90	8.13	8.20	NFV02	7.30	7.50	7.14
RTV57	8.10	8.07	7.84	NFV05	6.92	6.90	6.98
RTV58	7.24	7.45	7.72	NFV06	7.04	7.54	7.49
Test set				NFV21	7.51	7.11	7.08
RTV05	8.21	8.70	8.42	NFV23	8.27	8.57	8.39
RTV06	8.04	8.25	8.14	NFV25	6.73	6.81	6.68
RTV08	7.85	8.58	8.52	NFV27	6.34	7.21	7.21
RTV09	7.51	7.37	7.41	NFV43	8.40	7.71	7.61
RTV10	7.76	7.66	7.71	NFV47	7.05	7.42	7.56
RTV18	5.63	6.09	5.80	NFV48	7.63	7.44	7.40
RTV20	5.05	5.56	5.38	NFV53	8.44	8.57	8.19
RTV22	6.03	6.16	6.08				
RTV38	6.50	6.85	6.49				
RTV41	6.59	6.89	6.94				
RTV51	8.41	7.83	8.08				
RTV53	8.85	7.88	8.11				
Training set				Training set			
APV_WT	9.00	9.08	9.06	LPV_WT	9.00	8.99	8.97
APV02	8.34	8.39	8.27	LPV02	8.48	8.65	8.49
APV03	9.00	9.22	9.10	LPV03	8.47	8.64	8.54
APV06	9.70	9.60	9.69	LPV04	9.14	9.05	9.08
APV08	8.82	8.90	8.84	LPV05	8.64	9.00	8.88
APV11	9.00	9.04	9.06	LPV06	8.95	9.05	8.84
APV13	6.70	6.65	6.70	LPV07	8.57	8.37	8.54
APV14	7.58	7.38	7.38	LPV08	8.04	8.33	8.13
APV15	7.05	7.14	7.02	LPV11	9.76	9.30	9.62
APV24	8.61	8.69	8.59	LPV15	6.39	6.27	6.47
APV25	6.57	6.49	6.63	LPV24	8.98	9.14	9.13
APV26	7.24	7.22	7.25	LPV26	7.34	7.43	7.39
APV27	9.14	9.16	9.05	LPV27	7.56	7.56	7.58
APV30	6.11	6.00	6.17	LPV29	6.84	6.69	6.86
APV31	8.02	7.97	8.08	LPV30	6.33	6.18	6.25
APV32	8.56	8.64	8.55	LPV31	6.57	6.65	6.49
APV33	8.46	8.24	8.33	LPV32	7.58	7.60	7.58
APV34	7.24	7.42	7.41	LPV33	8.34	8.41	8.23
APV35	9.08	9.10	9.05	LPV34	7.09	7.41	7.21

APV36	5.74	5.85	5.67	LPV35	8.79	8.82	8.74
APV40	9.05	9.08	9.16	LPV36	4.84	4.89	4.78
APV44	9.01	8.77	8.85	LPV37	7.61	7.70	7.67
APV45	8.65	8.63	8.74	LPV41	8.79	8.82	8.89
APV47	8.82	9.00	9.09	LPV44	9.02	9.11	8.89
APV49	9.35	8.96	8.95	LPV45	8.87	8.88	8.73
APV50	9.01	8.94	8.95	LPV48	8.79	8.49	8.46
APV51	9.14	9.02	9.03	LPV50	8.14	8.40	8.37
APV52	9.05	9.00	9.04	LPV51	8.76	8.42	8.83
APV54	8.43	8.73	8.79	LPV52	8.36	8.08	8.37
APV56	7.08	7.33	7.27	LPV53	8.87	8.40	8.70
APV58	8.19	8.10	7.97	LPV54	8.41	8.59	8.69
Test set				LPV56	6.88	7.04	6.87
APV07	9.02	8.95	8.81	LPV58	8.74	8.60	8.68
APV12	8.52	8.21	8.22	Test set			
APV28	7.21	7.78	7.51	LPV01	8.79	8.76	8.68
APV29	6.51	6.97	6.88	LPV12	8.36	8.08	8.58
APV37	7.64	7.71	7.20	LPV13	7.50	8.26	7.70
APV41	8.30	8.07	7.74	LPV14	7.21	7.24	7.06
APV48	9.40	9.24	9.06	LPV25	6.34	7.14	7.36
APV53	8.90	8.77	8.85	LPV28	7.75	7.92	7.79
APV55	6.42	7.11	7.14	LPV40	9.02	8.52	8.51
				LPV47	8.56	8.52	8.69
				LPV49	8.76	8.67	8.51
				LPV55	7.98	7.68	7.76
